# Healthcare Providers’ Perceptions of Potentially Preventable Rural Hospitalisations: A Qualitative Study

**DOI:** 10.3390/ijerph182312767

**Published:** 2021-12-03

**Authors:** Andrew Ridge, Gregory M. Peterson, Bastian M. Seidel, Vinah Anderson, Rosie Nash

**Affiliations:** 1 School of Pharmacy and Pharmacology, College of Health and Medicine, University of Tasmania, Hobart, TAS 7000, Australia; G.Peterson@utas.edu.au; 2 Huon Valley Health Centre, Huonville, TAS 7109, Australia; b.seidel@huonmedicalgroup.com.au; 3 School of Medicine, College of Health and Medicine, University of Tasmania, Hobart, TAS 7000, Australia; Rose.McShane@utas.edu.au; 4 School of Health Sciences, College of Health and Medicine, University of Tasmania, Hobart, TAS 7000, Australia; Vinah.Anderson@utas.edu.au

**Keywords:** rural, primary care, preventable hospitalisation, avoidable, health literacy, access

## Abstract

Potentially preventable hospitalisations (PPHs) are common in rural communities in Australia and around the world. Healthcare providers have a perspective on PPHs that may not be accessible by analysing routine patient data. This study explores the factors that healthcare providers believe cause PPHs and seeks to identify strategies for preventing them. Physicians, nurses, paramedics, and health administrators with experience in managing rural patients with PPHs were recruited from southern Tasmania, Australia. Semi-structured telephone interviews were conducted, and reflexive thematic analysis was used to analyse the data. Participants linked health literacy, limited access to primary care, and perceptions of primary care services with PPH risk. The belief that patients did not have a good understanding of where, when, and how to manage their health was perceived to be linked to patient-specific health literacy challenges. Access to primary healthcare was impacted by appointment availability, transport, and financial constraints. In contrast, it was felt that the prompt, comprehensive, and free healthcare delivered in hospitals appealed to patients and influenced their decision to bypass rural primary healthcare services. Strategies to reduce PPHs in rural Australian communities may include promoting health literacy, optimising the delivery of existing services, and improving social support structures.

## 1. Introduction

Potentially preventable hospitalisations (PPHs), also referred to as admissions for ambulatory care sensitive (ACS) conditions, are used to measure the performance of primary healthcare (PHC) in Australia [1,2] and overseas [3,4,5]. In Australia, a PPH is defined in the National Healthcare Agreement as an ‘admission to hospital for a condition where the hospitalisation could have potentially been prevented through the provision of appropriate individualised preventative health interventions and early disease management usually delivered in primary care and community-based care settings’ [6,7]. PPHs account for 6.6% of all Australian hospital admissions, or almost 750,000 hospital separations per annum, with the incidence increasing by about 3% each year [8]. PHC quality and equity are often measured by PPH rates [6], although the ‘preventability’ of each condition is less well defined [9].

Australia’s health system is funded by a mix of public and private funding. The universal health insurance scheme, Medicare, provides free hospital treatment at public hospitals throughout the country and subsidises healthcare services provided by general practitioners (GPs) and some allied healthcare professions. Additional charges may be levied by providers; dental services are delivered outside the Medicare system, while ambulance services are provided without charge in some states (including Tasmania).

Multiple determinants interact at the individual, interpersonal, system, and environmental levels to influence the risk of PPH [10]. Previous studies of Australian community-based service providers have suggested limited access to PHC services and the patients’ perception of the urgency of their condition are strong predictors of PPH [11]. The urgency with which patients seek hospital care is often based upon a self-assessment of their condition, and this, in turn, may be a reflection of their health literacy [11,12]. Healthcare workers and patients often have differing views of the need for emergency hospital care [11].

Tasmania, the smallest state of Australia, has a geographically dispersed population that is older than the national average and has a higher prevalence of risk factors for poor health, such as smoking, risky alcohol consumption, physical inactivity, and obesity [13,14]. Socioeconomic risk factors, such as educational attainment, income, housing, limited access to services, and health literacy, are prominent risks for the Tasmanian population generally, and more so for populations outside urban areas [13,14].

Compared with urban populations, rural Australians experience generally poorer health, lower income, geographical isolation, and reduced access to services, all of which can increase the rate of PPHs [14]. Importantly, access to PHC represents more than health service availability. The concept of access incorporates dimensions of physical accessibility, organisational accommodation, affordability, and acceptability [15]. For patients from rural and remote communities, access barriers include unavailability of services, awareness of and ability to use a service, poverty, geographical and transport issues. In addition, ‘internal’ factors preventing service access in rural areas include patients’ fear of losing independence, stoicism, and a denial of their changing health status [16].

Previous studies have examined patients’ perceptions of PPHs [11,17,18,19,20], but the views of healthcare providers have been less frequently studied [11,17,21]. Studies recognising the views of healthcare providers other than hospital-based physicians and nurses are lacking, as is the focus on the broader rural adult population. The purpose of this study was to determine what factors healthcare providers, from a range of professions and workplaces, believe contribute to PPHs in rural Tasmania, Australia, and to identify possible methods of reducing PPH occurrence.

In the following sections, the methods used to identify and recruit participants, and the approaches to data collection, analysis, and identification of themes are presented. The themes developed are discussed, and implications for future practice and research are suggested.

## 2. Materials and Methods

Consistent with a pragmatist worldview [22], reflexive thematic analysis was selected as an appropriate analytical method to understand and construct themes from the data [21]. The 32-item COREQ checklist [23] was used to ensure rigour in the methods and analysis. An inductive method based on Braun and Clarke [24] was used to answer the following research question: according to relevant healthcare providers who have a lived experience of rural PPH, what are the drivers of PPH admissions, and can these admissions be prevented (if so, how)?

### 2.1. Setting and Participants

Purposive sampling ensured a variety of professions and experience levels from the southern Tasmanian (Australia) health system would be represented in the data [25]. Desired interviewees included medical, nursing, paramedic, and health-managerial professionals from a mixture of private and public practice. Department leaders, research coordinators, and liaison officers from the local Council, Tasmanian Health Service, and Ambulance Tasmania were initially contacted to assist in recruitment. Professional networks were used to find appropriate participants as needed.

Participants were doctors (*n* = 6), nurses (*n* = 4), paramedics (*n* = 2) or non-clinical health administrators (*n* = 2). Doctors were primarily employed in the emergency department (ED) of the Royal Hobart Hospital (RHH; *n* = 4) or as general practitioners (GPs) in the Huon and Bruny Island area (*n* = 2). Two of the doctors had recent or current experience working in both PHC and the tertiary setting. Nurses worked either in the RHH (*n* = 3) or as a community nurse (*n* = 1). Participant details are presented in Appendix A.

### 2.2. Data Collection

Informed consent was obtained in writing from all participants. Telephone interviews were conducted by two research team members experienced in qualitative data collection, during November and December 2020. A semi-structured interview design with an interview guide was chosen so that each interviewee could express their thoughts in a style and depth with which they were comfortable without being constrained by profession-specific jargon or questioning (Appendix B). Information and consent forms were distributed and collected electronically.

The interviews were digitally recorded and the audio files professionally transcribed verbatim. No participant names or identifying comments were included in the final transcript. An interview guide was used to ensure the semi-structured interviews covered all desired topics. During the interview, an ‘idealised’ patient from the rural Huon-Bruny Island area of southern Tasmania was used to focus the discussion.

Reflexive thematic analysis was used by one author (A.R.) to code, interpret and present key patterns in the data, using the methods described by Braun and Clarke [24,26]. The initial coding was descriptive and closely reflected the data before similar codes were arranged together. Core themes were developed from the grouped codes to help interpret the data. Using NVIVO (QSR International Pty Ltd., Doncaster, Australia), a coding check was performed on interviews to ensure coding consistency. Coding and thematic veracity were checked by two additional investigators (G.P. and R.N.). The reflexive journal and codebook were reviewed by an additional researcher (R.N.), and the researchers’ role in the research process was considered.

### 2.3. Ethics

Approval for the project was obtained from the Human Research Ethics Committee of Tasmania (Reference: H0018575).

## 3. Results

Most interviewees (*n* = 11) provided a definition of PPH in their own words. Definitions reflected the belief that some presentations and admissions to hospitals could be avoided by more appropriate and timely treatment in the community, such as the following:

*… broadly speaking, preventable hospital admission would be one that is a diagnosis or condition that could reasonably be treated out in the community but perhaps due to lack of resources or other types of backup, that the patients actually require admission to hospital …*

(313)

*… lots of chronic long-term illnesses that are probably not overly managed all that well in the community but end up [in hospital] …*

(316)

*… the various kind of conditions that people have and the ones that are specifically amenable to earlier intervention as a means of preventing hospitalisation …*

(325)

Every interviewee was able to provide at least one example of what they considered to be a PPH. Chronic obstructive pulmonary disease (COPD) was the most common condition considered a PPH (*n* = 7), with skin infections (including cellulitis; *n* = 6), diabetic complications (*n* = 5), dental problems (*n* = 4), heart failure, and mental health (*n* = 3) also cited. Other examples were not diagnosis specific, such as ‘chronic disease’, ‘wound dressings’, ‘intravenous antibiotics’, ‘palliative care’ and ‘falls’. Exacerbations of COPD were mentioned as being *not* preventable by one participant. Participants did not usually distinguish between long-term or antecedent risk factors and more immediate ‘triggers’ of PPHs, with their descriptions often intertwined. Definitions and examples were provided to ensure concordance between interviewer and interviewee understanding of PPHs.

Broad themes of patient health literacy, access to PHC services, and perception of hospital treatment were inductively developed to understand the data (see Supporting Information). Participants who gave a description of a ‘typical’ rural patient at risk of PPH described them as older, isolated, having poorer educational attainment, a higher comorbidity burden, and lower socioeconomic status.

*… the types of admissions that sort of stick in my mind more commonly are the elderly, more frail type people that have multiple comorbid illnesses, that have more social type issues for admission.*

(313)

*They’re older. They’re not particularly well educated. Often dependent on some form of government support, even if working. And often there are a number of complicating factors…*

(325)

### 3.1. Patient Health Literacy

The most common factor causing inappropriate healthcare service use raised by the health professionals interviewed was patient health literacy challenges. Wider population health literacy was believed to be poor but worse in rural or low socioeconomic status communities. Healthcare providers cited patients’ limited knowledge of their health as a common factor predisposing them to or causing PPH. It was reported that there was a poor understanding of what medical conditions were trivial, could be managed by rural health services, or warranted hospital management.

*… people just aren’t thinking about their own health enough and coming to emergency with really minor things… [patients should be] clear about… when they need to access ED and when they need to access GPs…*

(302)

*… what we might consider as a minor problem, to them is a major problem and they think it’s appropriate to go to emergency, so there’s partly an education thing about what’s minor and what’s not.*

(309)

Conversely, delaying presentation for an ACS condition until it required hospital intervention was another manifestation of poor health literacy. Rural populations and older patients were viewed as being more likely to delay presenting for healthcare.

*… rural patients are more likely to stay at home for longer, you know, wait longer before seeking medical care.*

(312)

*… people will wait until the point where it’s no longer getting better in their mind; it’s actually getting worse, and this is where they actually start to present to the hospitals.*

(324)

Rural patients with limited individual health literacy were believed to lack confidence in their ability to manage their health in the community and need reassurance during an acute medical episode. In addition, there is a lack of understanding of where to get this reassurance, especially after business hours.

*… [they] genuinely don’t know what to do, so they end up with us in an ambulance.*

(317)

*… I think people get frightened and they want to go to hospital sometimes*

(319)

The burden of navigating the rural PHC system, particularly for individuals with poor health literacy, was believed to be onerous and favoured hospital presentation. Patients have a clear understanding of how to access the hospital via the ED and/or ambulance services, so the simplest solution is frequently bypassing PHC.

*I don’t think people really know who to call, and it gets to the point where the only option they can do is call 000.*

(324)

Prolonged disengagement with the health system was viewed as detrimental to healthcare, mainly through a reduced opportunity for educative and preventative service delivery.

*… their condition isn’t managed correctly, and again that goes back to just our population, the lack of health literacy and socioeconomic factors prevent them from accessing their good community care …* *they don’t feel like they’re unwell so they’re not managing their condition, that lack of awareness of what can happen if they don’t* *…*

(316)

*… long term poor chronic disease management,… is often due to patients not doing the right thing and not looking after themselves and not going along to see the GP, unfortunately.*

(312)

### 3.2. Patient Access to Primary Care

Access to PHC services contained sub-concepts of GP appointment availability, logistical issues associated with using PHC services, and financial constraints. The inability to obtain an appointment for PHC services within the desired timeframe was seen as a common reason rural patients presented to the hospital without first engaging with PHC services. An urgent ‘on the day’ appointment may not have been available, or the condition could have been treated in the community if alternative health services were utilised.

*I think some people find it difficult to access GP services… certainly in some areas access to GPs is poor. They may have a limited number of on-the-day appointments.*

(315)

*… people are waiting four weeks to get into a GP here and then they can’t often see the same one and if they’ve got a chronic illness…*

(316)

*People don’t conveniently have their medical issues during business hours, it happens after hours.*

(317)

Lack of transport was considered especially problematic in rural locations where distances between the patients’ residences and medical care are relatively greater. Transport options are further restricted outside normal business hours when public or social transport is less accessible. Accessing transport and medical care via the ‘free’ ambulance service in Tasmania was one method used to overcome travel and cost barriers.

*… they can’t just walk out their front door and jump on a bus. If these people don’t drive, they don’t have the social networks to actually get them there readily.*

(317)

*… arguably with ambulance, access to ambulance with no cost, there’s an incentive that makes for very good access to that sort of treatment and that sort of care. And from the perspective of a person who doesn’t have many resources or may be isolated, and doesn’t want to make the multiple trips associated with diagnostics, it could potentially present as a no-brainer to seek help in that regard.*

(325)

Even when private transport is available, patients from rural communities face the logistical barrier of longer travelling times to and from local and hospital services. The likelihood of hospital admission increases as a result of the higher degree of suspicion and caution applied to rural patients.

*… if someone comes to ED from a rural area and it’s something quite minor that I would usually say, ‘Look, I don’t even want to triage you. I think you could probably go to your GP,’ if they’re from a rural area, I’d be less likely to do that, because it’s just they’ve driven all the way in, it’s a big drive back.*

(302)

Rural patients faced financial barriers when seeking to access PHC. Accessing preventative or chronic-disease services is given a lower priority than other financial demands faced, or the value of such health services was not appreciated.

*… I think it probably boils down to two main reasons: one is that most people in Tasmania find it hard to afford a private billing GP*

(314)

*… patients in rural areas identified as being lower socioeconomic, their understanding is that for them to actually go to their GP is an out-of-pocket expense, and that money is somewhat better spent, in their mind, on the requirements of everyday living.*

(324)

Medical and allied health services often advise patients to seek hospital treatment if their condition does not improve or PHC services are unable to provide ongoing care. This was viewed as a frequent, ‘default’ recommendation that was more likely to occur after hours.

*I just find Healthline, I think they seem to increase our presentations, because their protocol [results in them advising] ‘You better go to ED,’ seems to happen very regularly.*

(302)

*Again, I have experience of people rocking up and saying, ‘Well, I rang Call the Doctor, and they said, ‘Come straight in.’’*

(308)

Healthcare providers who *could* contribute to patient care in the community (e.g., social workers, occupational therapists, physiotherapists, psychologists, dentists) were not being fully utilised. The interviewees suggested barriers to involving other care providers include a lack of awareness of services available, restrictions on the scope of practice, and limited service availability.

*… you either forget about it or you never hear about it in the first place or two years later you realise there’s been some service running you’ve never heard of.*

(315)

*[paramedics] do have antibiotics that we can administer for patients for… acute otitis media, if we in the first instance gain an agreement with the GP for a follow-up appointment and the GP for continuations of that prescription because we are limited to one singular dose only.*

(324)

### 3.3. Perceptions of Hospital Care

A belief that patients may view the hospital as a place where comprehensive primary care can be provided was shared by some interviewees. The accessibility of a wide range of medical specialists, testing facilities, and treatments was believed to make bypassing general practice attractive to rural patients.

*A lot of people, I think, use [the hospital] as their GP, really.*

(314)

*People just want to be sorted straight away … I think there’s a perception that if you come to emergency, you’re getting everything at once.*

(302)

*There’s probably a culture in Tasmania… of substituting ED and hospital presentations for access to primary care … why go to your general practitioner and they write you up for an X-ray, when you can front up to the ED and if you need it, you can get a chest X-ray, you get a CT, full panel of bloods and all sorts of stuff done.*

(325)

Time and scope of practice limitations in PHC were considered to result in patients being treated for minor ailments in the hospital. Interviewees raised concerns that general practice appointment length is not sufficient to address all patients’ needs, and financial constraints make minor procedures less attractive for GPs to deliver, causing patients to seek these services in the ED.

*… in my experience in ED, people needing suturing and small procedures like incision and drainage, that sort of thing, now have to get admitted to hospital because GPs don’t seem to be doing that stuff… they don’t have enough time.*

(302)

### 3.4. Suggested Solutions

Increased patient engagement and involvement with their own healthcare was considered to be one method of improving health literacy. Interviewees highlighted that continuity of contact with a regular community-based provider could improve health literacy, encourage health-seeking behaviour, and promote adherence to long-term interventions.

*… one of the solutions obviously is better education about how to self-manage your chronic condition.*

(302)

*There just seems to me to be lack of preventative health and I don’t know if that goes back to as far as early prevention at schools, more intervention in communities, more education…*

(316)

*… I think people who see their GPs more often are more engaged, have better understanding of their chronic conditions, might be more likely to seek help earlier perhaps.*

(315)

Access to after-hours GP services was frequently discussed, and adequate funding of services was suggested to reduce PPHs. A stand-alone GP service that bulk-bills patients (i.e., no cost to the patient) and provides accessible after-hours care was a proposed way of reducing PPHs caused by poor after-hours access to GPs.

*… I definitely think that better resourcing for out of hours general practice would reduce emergency department visits and hospital admissions…*

(312)

*… better access to bulk-bill GPs with minimal wait times for appointments would certainly stop people turning up.*

(314)

*… I mean GP Assist are brilliant. Without them I think we would really struggle, so I think that ongoing funding and provision of GP Assist is essential…*

(309)

Those interviewed suggested that ensuring patients were followed up at critical points in their health management could prevent PPHs. A structured follow-up procedure to ensure certain patient groups had an arranged appointment for follow-up or were regularly monitored was believed to be lacking.

*… if the GP could just follow them up a bit tighter and make sure they have an appointment there for them to come back, they knew someone was going to review them if they were still well but just a bit unsure, that would save a lot of re-presentations*

(314)

Improving awareness of and simplifying access to services were suggested as solutions to fragmentation of care in rural communities. Better integration of existing Medicare-funded service providers, such as nurse practitioners, could also reduce PPHs.

*… disseminating information about [the Community Rapid Response Service] service, and maybe expanding it… to me that sounds like a great thing, where a patient can come to a clinic, be seen, and then be treated in their own home.*

(302)

*… [ideally] if you’re in a more regional or rural area, you’d have basically easy referral structures for social workers and other allied health professionals and also making sure that … there’s a means by which the GPs or other providers can actively engage services that—to which patients are entitled to make sure that they’re supported at home.*

(313)

*… I think if you’ve got an opportunity to create a standard bulk billing non-financial out-of-pocket for patients, then in rural areas you may actually see more people wanting to access medical care.*

(324)

## 4. Discussion

This study sought to understand what factors healthcare providers believe are associated with PPHs in rural communities. Published, qualitative studies of healthcare providers’ views of PPHs exist from overseas [19,27,28,29,30], but consideration of these views in the wider Australian, and specifically rural, context is less common [11,17,21]. Participants in this study defined PPHs in a way consistent with Australian standards [6], adding confidence to the findings.

Several of the factors identified in this study have previously been identified as significant drivers of PPHs in other Australian studies [11,17,31]. Our study shows that the perceived root causes of PPHs among rural Tasmanian adults were consistent across a wide range of hospital, community, and managerial healthcare providers. Themes of health literacy challenges, access to healthcare services, and perception of hospital convenience were commonly expressed by all participants. Sub-themes used to further describe these beliefs were significantly interrelated. Health professional insights and solutions for reducing PPHs were also analysed and presented.

There was little variation in the range of views expressed between professions. Interviewees who primarily worked in a hospital had a greater focus on health literacy and the inability to access PHC services. The GPs interviewed believed lack of access to services of all types and the limited range of services in the community were major causes of PPHs. Understanding the views of the broader rural health workforce, its service gaps, and potential capacity may be useful for future workforce planning and resources allocation [32].

### 4.1. Health Literacy

Health literacy challenges among rural patients, and their immediate social network was consistently identified as a major factor in causing PPH risk. An individual’s health literacy encompasses health-related decision making, the ability to seek health information, and being responsible for controlling one’s own health [33]. A belief that patients do not seek help at an appropriate time has been confirmed elsewhere as an independent risk factor for PPHs [31,34]. The effectiveness of providing patients with disease-specific educational interventions is considered to be of little value in reducing PPHs [20]; a broad-based approach to improving health literacy may be more appropriate. The role of location and isolation in reducing patient health literacy was described by interviewees and aligns with healthcare providers’ views from other settings [35,36,37].

The perception that it is ‘too late’ to address the health literacy of older patients was noted by one participant in this study, suggesting less importance may be given to improving health literacy among older rural patients. A contemporary definition of health literacy recognises the importance of patients’ social supports and the health service they are attempting to access as contributors to overall health literacy [38]. It is important that health services consider how responsive to their patient’s health literacy needs they are in their practice, and how their service might be improved to support all their patients, regardless of sociodemographic characteristics [39]. To date, there have been too few studies in rural Australia that explore primary healthcare’s response to health literacy challenges among their patients, although evidence is emerging that improving organisational health literacy is a realistic goal for most non-urban organisations [40,41].

Patients living alone or in geographically or socially isolated areas were believed to have fewer opportunities to access a support network wherein health literacy skills may have been ‘distributed’ [42]. The relative contribution of health literacy resources by the patient or their support network was not analysed in this study, but there is evidence that fewer cumulative health literacy resources available to patients can cause inappropriate (non-)use of healthcare services and thereby increase the risk of PPHs [29,36,37,43]. This corresponds with Australian data showing those living alone had fewer social supports [44]. The effects of social isolation, especially in older, rural populations, have been noted in other studies [36,45].

Healthcare providers in this study believed a patients’ perception of the urgency and seriousness of their condition was the most important factor when deciding to seek hospital treatment. It seems clear that while healthcare providers do not believe many conditions require hospital treatment, patients do [11,12,33]. A recognition by healthcare providers that patients are genuinely concerned about their health and are not ‘to blame’ for PPHs may be useful when considering interventions [43]. Addressing patients’ knowledge of what is appropriate to treat in the community and when to seek medical help could be a more appropriate response and could be assisted, for example, by an improved national telephone support line [46]. Delaying treatment, patient independence and stoicism, are also factors to consider when considering PPH interventions as these phenomena may be more prevalent in rural populations [16,47].

The link between poor health outcomes, health literacy challenges and rurality is not straightforward; sociocultural factors, rather than geographical location per se, have been suggested as the mechanism by which location and health literacy are linked [48]. Identifying what these local risk factors actually are may help in the design of interventions aimed at improving health literacy and reducing PPH rates in rural communities [49].

### 4.2. Service Accessibility

Access to rural PHC and the complexity of the health system was noted by most interviewees to have a large influence on PPHs. Availability of timely and affordable GP appointments was believed to be a powerful driver of PPHs and has been identified by hospital workers in other rural settings [17,31,50]. Deficiencies in other dimensions of ‘access to care’ (viz. geographic accessibility, organisational accommodation, affordability, and acceptability) were considered to increase the risk of PPHs in rural populations in this and other studies [50,51]. The disparate healthcare needs of rural populations may be better served by focussing improvements on the ‘non-supply’ dimensions of healthcare access.

Financial barriers to PHC services and reduced home or after-hours services have elsewhere been linked to PPHs [11,47,50]. For GP services in Tasmania, there was a decrease in no-cost (‘bulk-billing’) rates and an increase in out-of-pocket costs from 2012–2013 to 2018–2019 [52]. Not surprisingly, healthcare providers, here and elsewhere, suggest that improved support for bulk-billed GP consultations and other general practice services (such as preventative care, home visits, minor procedures, and follow-up services) would reduce the financial barriers to PHC treatment and allow more ACS conditions to be identified and managed appropriately outside hospitals [11,17,20,31]. Several GPs noted here that the provision of comprehensive care in a rural population is incompatible with the current Medicare Benefits Schedule (MBS) payment structure (‘fee for service’) in Australia.

The status of ‘GP supply’ as the main driver of PPHs in rural Australia is not universally accepted. In a large study linking qualitative interviews and routine health data, Falster et al. [53] suggested an individual’s socioeconomic status and health characteristics were more important drivers of PPHs than population-level and GP-supply metrics. Other Australian qualitative studies suggest access to GPs is both timely [54] and does not act as a driver of PPHs [11]. Therefore, it may be that PPHs follow a gradient in *health*, as opposed to a gradient in *healthcare* [53]. This requires further research.

The perception that after-hours services ‘default’ to referral to hospital for ACS conditions was recorded here and is supported by data showing expenditure on after-hours specific MBS items increasing significantly since 2013–2014 without a corresponding decrease in low-acuity ED presentations [55]. An appropriate model for after-hours deputising services in rural communities, as well as its impact on PPHs, is unclear. Retaining telehealth services introduced during the COVID-19 pandemic may be an additional way of encouraging healthcare participation and delivering chronic disease management in this population [46].

The beneficial role of long-term engagement with PHC has been previously noted, with interventions to manage lifestyle, comorbid or social variables, promote early diagnosis, and improve health literacy ‘30 years prior’ suggested as intervention methods [56].

### 4.3. Convenience of Hospital Care

The convenience of hospital treatment was put forward as strongly influencing rural patients’ decision to bypass PHC. Prompt, comprehensive, and ‘free’ hospital services (viz. ambulance transport, medical care, accommodation, testing, and procedures) were considered to be responsible for patients favouring ED over community services. Patients with PPHs acknowledge that the ‘convenience of hospital treatment’ does influence their propensity to attend hospital and is evidence that patients self-select the most efficient mode of care for them [18,45]. Interviewees identified a perceived local cultural norm of ‘if you get crook, you go into hospital’ which, along with other factors, lowers the threshold for hospital use; this has been observed in other populations [29], but it is unclear how or why patients arrive at this conclusion. Cheek et al. [18] found the perceived need for technical expertise was a significant driving force for patients by-passing PHC. Processes to assist patients in correctly identifying situations where presentation to the hospital is appropriate will likely incorporate aspects of increased health literacy and access to and understanding of the full gamut of community-level health services.

Interviewees believed ‘iatrogenic’ PPHs may be a result of non-specific recommendations (e.g., ‘come back and see me’ or ‘go to the hospital if things do not improve’) or poor coordination of follow-up services. There are few data on how frequently this results in hospitalisation. Enhanced multidisciplinary provision by colocation of services has been promoted as a means of improving PHC services [46]. As suggested by interviewees here, simplified appointment coordination and greater continuity of care may make community care more attractive and available while simultaneously reducing PPHs.

## 5. Limitations and Future Directions

As this was a qualitative study, its results cannot be generalised to other settings. This study does, however, identify themes associated with PPHs that have been reported in the Australian literature [57]. The effects of social isolation, especially in older and rural populations, have been noted in other studies [36,45], but the specific mechanisms involved, and appropriate interventions are not yet clear; this requires further research.

## 6. Conclusions

This qualitative study collected and analysed the perceptions of healthcare providers regarding the underlying causes of PPHs from a rural population. Long-standing health literacy challenges, the relative inaccessibility of local, timely, and affordable healthcare, and the perceived convenience of hospital-based care were identified by the participants as factors contributing to PPHs. Addressing these underlying issues was seen by the health professionals as a way to reduce PPH rates.

The interplay of health literacy and access themes developed in this study is similar in complexity and interconnectedness to those identified in other research studies among rural healthcare providers [11,17,43,51]. Any potential improvement, service redesign, and policy changes aimed at reducing PPHs will require consideration of the views of both patient and provider. An emphasis on clinical or supply-focussed interventions alone fails to recognise all the local-level drivers of PPH in rural areas. A broad-based approach to health literacy education and the health literacy responsiveness of the health system should form part of the response to the issues identified in this study. Incorporating all aspects of ‘access’ to health and community services offered in rural Tasmania will require a coordinated approach from all levels of government and providers of services in the area.

## Data Availability

Deidentified data (interview transcripts) were available to all authors throughout the project.

## Appendix A

**Table A1:** Healthcare provider interviewee details.

Participant Identifier	Profession	Position	Location
302	Registered Nurse	Lecturer; Nurse	Emergency Department, RHH
308	Registered Nurse	Nurse	Emergency Department, RHH
309	Doctor	GP	Huon-Bruny Island area
312	Doctor	Emergency Specialist	Emergency Department, RHH
313	Doctor	Emergency Specialist	Emergency Department, RHH
314	Doctor	Registrar	Emergency Department, RHH
315	Doctor	Emergency Registrar & GP	Emergency Department, RHH & Community GP
316	Registered Nurse	Nurse	Emergency Department, RHH
317	Paramedic	Intensive Paramedic	Hobart area
318	Doctor	GP	Huon-Bruny Island area
319	RN	Associate Nurse Manager	Huon-Bruny Island area
320	Health Administrator	Managerial	Department of Health, Hobart
324	Paramedic	Intensive Care Paramedic;Extended Care Paramedic	Hobart area
325	Health Administrator	Managerial	Department of Health, Hobart

Abbreviations: GP—general practitioner; RHH—Royal Hobart Hospital.

## Appendix B. Semi-Structured Telephone Interview Guide

### Healthcare Providers’ Perceptions of Potentially Preventable Hospitalisations: A Qualitative Study

Method: Semi-structured telephone interview

Location/Time/Date: T.B.C.

Interviewer: V.A. or A.R.

Introduction:

- Welcome to telephone interview and thanks
- Any initial questions (e.g., risks that may be posed)

Project Description:

- Healthcare providers’ perceptions of potentially preventable hospitalisations:
- a qualitative study
- *i.e., ‘seeking to understand healthcare providers’ perspectives on (potentially avoidable) hospital admission.’*
- Further explanation of study (if required)

Discussion Points:

- What are the characteristics of a typical PPH patient?
- What are the distal causes of PPH?
- What are the proximal causes of a PPH?
- How could PPH be avoided, minimised or addressed (if not covered by above)?

Concluding Comments:

- Potential for further contact for follow up interview
- Opportunity for questions

Thank participant
